# Embedding equitable placement opportunities in undergraduate science degrees

**DOI:** 10.3389/fmed.2025.1713179

**Published:** 2025-12-15

**Authors:** Dominic Wiredu-Boakye, Shalinee Dhayal, Susanne Smith, Musarrat Maisha Reza

**Affiliations:** 1 Department of Clinical and Biomedical Science, University of Exeter, Exeter, United Kingdom; 2 Department of Health and Community Sciences, University of Exeter, Exeter, United Kingdom

**Keywords:** transferrable skills development, job-hunting and employability training, equitable access to placement opportunities, reflective assessment, institutional support, employer partnerships, widening participation, awarding gaps

## Abstract

Work placements is widely recognized as critical to preparing workforce-ready graduates across the science and health professions. Yet, in non-accredited science programmes, access to meaningful work experience often depends on students independently sourcing placements. This model privileges students with financial resources and professional networks while disadvantaging students from lower socioeconomic or international backgrounds, reinforcing structural inequities in higher education. This Perspective article argues that embedding structured, equitable, and assessed work experience into non-accredited biomedical and health sciences degrees is both a moral imperative and a strategic necessity for universities. Drawing on examples such as the University of Exeter’s Professional Training Year and Singapore Management University’s embedded internship model, we demonstrate how inclusive, scalable frameworks can bridge the gap between academic learning and professional practice. These approaches highlight the benefits of integrating reflective assessment, institutional support, and employer partnerships to strengthen student outcomes and confidence. We propose seven key recommendations to guide future practice: (1) establish joint industry and academic curriculum steering committees; (2) embed transferrable skills development within science curricula; (3) provide job-hunting and employability training; (4) ensure equitable access to placement opportunities; (5) deliver structured support to enhance student placement experiences; (6) assess work-based learning through reflective and practical outputs; and (7) align policy reforms with inclusive research and placement opportunities. Embedding equitable work placements at scale will not only improve employability and readiness for healthcare and research roles but also advance widening participation, close awarding gaps, and cultivate a socially mobile, resilient graduate workforce.

## Introduction

In the current UK Higher Education landscape, the need to produce workforce-ready graduates is more pressing than ever. Universities are under increasing scrutiny regarding the preparedness of their graduates, with some policymakers arguing that graduate outcomes should be used as a metric to determine whether universities are permitted to raise tuition fees (1). Universities have responded to this by designing degree programmes that focus on equipping graduates with generic skills that would ultimately make them more employable.

It is now widely accepted that employability also encompasses career management skills and various forms of capital such as identity, social, and cultural capital that enable graduates to navigate and thrive within complex labor markets [(2, 3)]. Crucially, these intangible skills are typically developed through work experience. To this end, science degrees accredited by regulatory bodies such as medicine, nursing, and certain biomedical science programs embed work experience in the form of clinical placements as a mandated component. In the UK, these regulatory bodies include the Health and Care Professions Council (HCPC), the General Medical Council (GMC), and the Nursing and Midwifery Council (NMC). Accreditation involves evaluation of programs against defined standards for the purposes of quality assurance and enhancement (4). As a result, students on accredited programmes benefit from a structured approach to practical learning, ensuring all students on the programme gain work experience as part of their educational journey.

Employers’ emphasis on practical readiness has also fueled the rise of higher-level apprenticeships, which embed structured work-based learning within permanent employment. Between 2024 and 2025, the number of Level 6 and Level 7 apprenticeships, which are equivalent to undergraduate and master’s degrees respectively, increased by nearly 9%. This represents over 18% of all new enrollments in England.

Non-accredited degree programmes offer universities greater curricular flexibility, allowing rapid adaptation to new scientific developments, technological advances, and evolving workforce needs (5). A recent UNESCO report highlights how these decentralized and market-focused UK degrees are exemplary in the way they provide routes to students to acquire knowledge and skills at different life stages and support learners with widely-ranging personal circumstances (6). Importantly, these programmes allow learners to tailor their education according to their individual interests and career aspirations, rather than being limited by the requirements of professional accreditation bodies. For example, a biomedical science student might choose to take modules in business management or Spanish, thereby broadening their skill set beyond the traditional boundaries of the discipline. This agility is especially valuable in the dynamic bioscience and health technology sectors, where emerging technologies continually reshape graduate skill requirements. Freed from accreditation constraints, non-accredited degree programmes can pilot innovative teaching methods, introduce future-focused modules, and respond promptly to feedback from students and employers (7).

On the other hand, due to the lack of a regulatory body dictating the inclusion of work experience in non-accredited science degrees, in many of these programmes, work experience placements are relegated as optional or extracurricular. Crucially, it is often incumbent on the student to secure these work experience placements alongside their degree.

Employers often prioritize relevant work experience when recruiting graduates. A 2024 CBI Economics survey found that 42% of UK employers valued vocational experience gained during a degree more highly than degree classification or institutional prestige (8). Likewise, research consistently shows that students value opportunities to develop employability skills and engage with employers during their studies. For students from lower socioeconomic backgrounds, higher education acts as a catalyst for advancing their social and economic status (9–11). At the policy level, governments increasingly position employability at the center of higher education, viewing the cultivation of a highly skilled workforce as essential for attracting foreign investment (12, 13). In this context, it can be argued that even non-accredited science degrees have an obligation to provide graduates with relevant work experience which in turn will enable them to thrive in the job market. Relying on students to independently secure work experience placements, often as optional, non-timetabled activities does not fully address the responsibility of higher education institutions to support students’ professional development.

## The hidden cost of non-timetabled optional work experience

**FIGURE 1:**
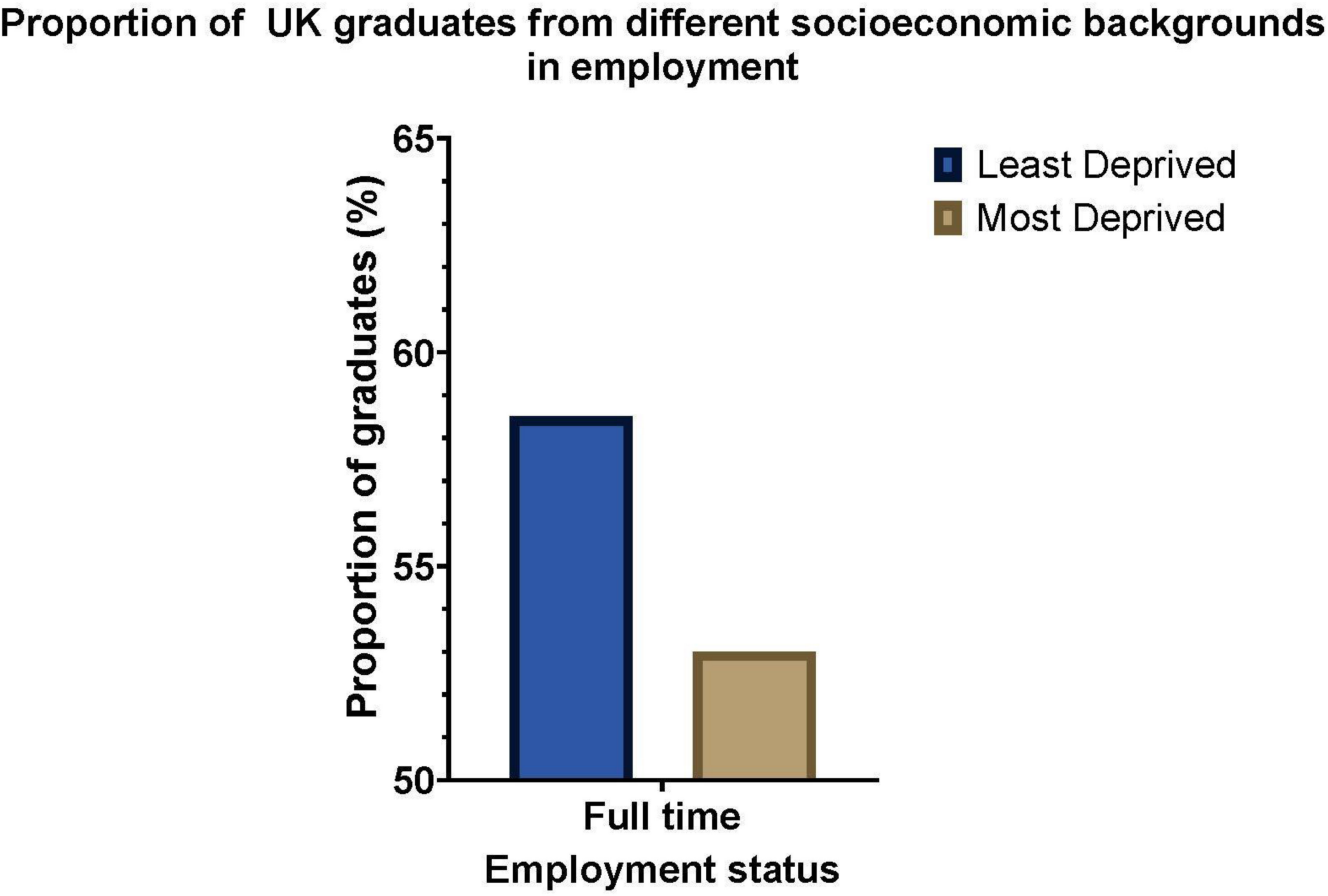
Employability outcomes for graduates from the most deprived (POLAR 1–2) and least deprived (POLAR 4–5) backgrounds. POLAR 1 represents areas with the highest levels of socio-economic disadvantage, while POLAR 5 represents areas with the lowest. Data sourced from the Higher Education Statistics Agency (HESA) report for 2021–2022.

Crucially, non-timetabled work experience placements disadvantage students from lower socioeconomic backgrounds who have to balance securing placements with their part-time jobs, caring responsibilities and academic commitments (14, 15) (Figure 1). This is especially true if these work placements are short-term and unpaid. These students often lack the Identity Capital (How a graduate sees themselves in relation to their future career and how much effort they put into preparing for their future career), Social Capital (The network of relationships and connections that help graduates find job opportunities and succeed in the workforce) and Cultural Capital (The knowledge, attitudes, and behaviors that are valued by the workplaces graduates want to join) to access high-quality work placements related to their degree (3). Due to limited access to the social networks associated with various forms of capital, students from lower socio-economic backgrounds often encounter repeated rejection when seeking work (Figure 1). This can lead to a diminished sense of belonging within the job market they aspire to enter, impacting their identity capital further.

Consequently, non-accredited science degrees risk becoming arenas where learners from disadvantaged backgrounds observe social inequality unfold, as their more affluent peers are able to seize career-defining opportunities that remain out of reach to them (16). There is a common narrative that the process of job searching and coping with repeated rejections fosters resilience (17–19). While this may hold true for some, it overlooks the significant financial and personal challenges faced by students from lower socioeconomic backgrounds. For these students, the additional burden of managing multiple rejections while completing a demanding science degree can become overwhelming. As a result, some may give up seeking opportunities altogether, ultimately graduating with limited labor market prospects, often not much improved from their pre-degree position.

For some international students, work experience placements may be largely inaccessible as a result of visa restrictions. Currently in the UK, international students on a student visa can only undertake work placements if these are an “assessed and integral” part of their degree programme; extracurricular or optional placements are limited to part-time hours during term time, and full-time only during vacations. These restrictions make it difficult for international students to access the same range of work experience opportunities as home students, particularly when placements are not embedded in the curriculum (20).

## Exploring models of best practice

In 2021, the UK Department for Education commissioned a review of literature on work experience placements in higher education since 2010. A key objective of this review was to examine strategies universities employ to promote inclusive participation in such placements. Sadly, the review found no information directly addressing this specific question (14). Below, we highlight examples of best practices adopted by select universities that answer this question.

### Case study 1: the Professional Training Year (PTY) at University of Exeter, UK

The University of Exeter’s Professional Training Year (PTY) is an optional 9 to 12-months credit-bearing industrial placement undertaken between the second and final years of the BSc Medical Sciences degree (now Biomedical Science). The aim is for students to gain practical experience of undertaking a research project in diverse settings, including NHS hospitals, research laboratories, biotechnology and pharmaceutical companies, government health agencies, and NGOs.

The PTY’s effectiveness stems from its structured and inclusive design. All students are first encouraged to source their own placements. To support this effort, a list, of vetted opportunities arranged by placement academic leads is provided. The university’s Career Zone also provides employability support through helping with sourcing various placements, providing guidance on writing CVs and applications as well as help with interviews. This approach helps to alleviate the pressures of finding a placement for students with limited identity, social, or cultural capital.

Between January and March, when most PTY placement applications are open, academic tutors run timetabled small group sessions and individual meetings to offer tailored support and financial advice to students. The academic tutors facilitate group activities that encourage students to identify and discuss the transferable skills they have gained during their degree that can strengthen their PTY applications. Students also receive peer mentorship from former PTY students throughout the application process (21), including at least two formal meetings during the year.

The written student feedback consistently demonstrates that as well as meeting the key expectation for students to gain practical work experience, students also feel increased confidence, clearer career goals, and a stronger sense of belonging in scientific or healthcare environments, reflecting gains in identity capital. Many return with academic publications [see Section “Acknowledgment” in (22) and Section “Author’s declaration” in the following doctoral thesis (23–25)], conference presentations, or job offers, demonstrating enhanced cultural and social capital. Similar benefits have been reported by the University of Leeds, which has also succeeded in widening access to placements regardless of socioeconomic background (26). Importantly, integrating this placement opportunity within the degree programme ensures compliance with UK visa regulations, making it accessible to international students.

#### Critique

The year-long pause can create challenges in maintaining academic continuity and may not suit all students, particularly mature students with financial or personal constraints such as caring responsibilities. This was evident in Divan et al’s report where only 4% of mature students took up work placement opportunities compared to 11% of young students. This challenge becomes more acute when the work placements are unpaid. Literature on work-integrated learning highlights the need for continuous and scaffolded support, which the University of Exeter addresses well but could be further enhanced through targeted mentoring for students who face disadvantages due to lower social capital.

### Case study 2: Singapore Management University’s (SMU) integrated work placement model in Singapore

Singapore Management University (SMU), Singapore, offers a compelling case study in the successful integration of work placement within undergraduate education through its mandatory, credit-bearing internship programme. All students are required to undertake at least one internship to graduate. Students apply for positions of interest from a curated database of opportunities sourced through SMU’s extensive industry partnerships which allows them to continuously apply academic knowledge in professional contexts, develop workplace-ready skills, and build meaningful industry networks through their internships This approach ensures students graduate with not only theoretical proficiency but also practical experience and a clearer sense of career direction (Identity capital) (27). Many students complete multiple placements, an average of 2.8 internships per student, with 79.9% completing more than one, enabling them to apply academic knowledge in diverse professional contexts and develop both technical and soft skills.

The success of this model is reflected in SMU’s outstanding employability outcomes (28). According to the 2024 Joint Autonomous Universities Graduate Employment Survey, 89.8% of SMU graduates secured employment within 6 months of graduation, with 83.4% entering full-time permanent roles. Notably, 31.6% of graduates reported gaining their jobs directly through internships undertaken during their studies (29, 30). SMU’s strong industry ties across sectors such as finance, law, technology, and consulting further reinforce the effectiveness of its embedded placement model.

While the survey does not disaggregate data by socioeconomic or other backgrounds, SMU’s universal, credit-bearing structure ensures equitable participation by making internships a funded, integral part of the curriculum rather than an optional or self-sourced activity.

For universities seeking to close employability gaps, particularly for students from underrepresented backgrounds, SMU’s model offers a roadmap for embedding equity, relevance, and career readiness into the fabric of higher education.

#### Critique of this model

The intensity of juggling multiple internships alongside academic requirements can place significant pressure on students, potentially disadvantageous to those balancing employment or caregiving responsibilities. Furthermore, while SMU boasts high employment rates, it would be interesting to investigate the depth of learning achieved across different placements. Without rigorous assessment of placements and reflective practice, short internships risk becoming superficial experiences rather than meaningful professional development.

## Reimagining science degrees with embedded placements

Drawing from current practice and the authors’ own institutional experiences, we offer a set of strategic recommendations for higher education institutions to reimagine undergraduate science education with equitable, embedded placements at its core. The framework we propose is below.

### Set up joint industry and academic curriculum steering committees

Embedding work experience meaningfully requires curricula that are responsive to labor market needs and real-world skill demands (31). Institutions should establish steering committees that include academic leads, clinical and industry professionals, employer partners, and student representatives.

These committees should meet regularly to review and co-develop programme content, ensuring that graduate attributes, module outcomes, and assessment strategies remain aligned with the evolving needs of healthcare, research, and technology sectors (32). Universities should formalize these relationships through placement learning agreements or memoranda of understanding, outlining roles, responsibilities, and outcomes for all parties (33). Careers services should support and equip academics to integrate employability and placement activities more extensively into core curricula, giving all students structured opportunities to participate.

Such collaboration also strengthens placement pipelines. Employers engaged early in curriculum design are more likely to invest in offering placement opportunities, mentoring, or even co-teaching (34). For students, this ensures a clearer connection between what is taught in-class and what is expected in the workplace. For universities, this can enhance employer relationships, graduate destination data, and curriculum relevance. For the industrial/healthcare partner, this presents an avenue to have an academic partner to facilitate staff life-long learning. This is particularly important in the context of the highly anticipated UK higher education funding reform (35).

### Teach transferrable skills as part of science curricula

Healthcare and research environments demand more than technical competence. They require graduates who can communicate effectively, work in teams, demonstrate empathy, manage time, and adapt to ambiguity [(36, 37)]. However, transferrable skills are often assumed to develop organically rather than taught explicitly within science programmes (38).

Universities should embed professional and interpersonal skill development into the curriculum, beginning in the first year and scaffolded across the degree (39). Techniques may include group projects with peer assessment, interprofessional simulation-based learning, reflective writing, and role-play scenarios (40, 41). Modules on communication in healthcare, ethics in healthcare and research, emotional intelligence, or leadership in science can complement laboratory and theory-based modules. By embedding these skills, universities prepare students not only to secure placements but to thrive in them (42).

### Embed job-hunting skills development

One of the most overlooked challenges students face is how to “market” themselves to potential employers, particularly when they lack prior experience, connections, or familiarity with the hidden curriculum of professional recruitment. Higher Education Institutes (HEIs) must take proactive responsibility for demystifying these processes. Career-readiness content should be embedded into core modules. This includes teaching CV writing, crafting cover letters, preparing for interviews, navigating professional etiquette, and understanding sector-specific application processes (43). Sessions should also address how to cope with rejection, recognize bias, and manage imposter syndrome especially for students from minoritized or first-generation backgrounds (44–46).

By equipping all students with the tools to access opportunity, universities can work toward mitigating disparities in placement outcomes and reduce the burden currently placed on self-navigation and informal networks.

### Ensuring equitable access to placement opportunities

When supporting students in their search for placements, it is essential to remove barriers that may disproportionately affect those from disadvantaged backgrounds (47). This can be achieved by widening placement listings to include a variety of sectors, locations, and flexible or remote roles, ensuring that opportunities are not limited by geography or travel constraints. In addition, financial accessibility must be prioritized by highlighting paid placements wherever possible and providing clear information on bursaries, hardship funds, and travel or accommodation support to ensure that no student is excluded due to cost.

### Providing structured support to enhance placement experience

As explained in Section “Case study 1: the Professional Training Year (PTY) at University of Exeter, UK” above, introducing structured triadic mentorship, linking the student, an academic mentor and a workplace supervisor throughout the placement period provides ongoing feedback, guidance, and tailored support is important in supporting placement students. Regular goal setting, review meetings, and formal progression reports help create a scaffolded learning journey that supports students’ growth and confidence (48). Embedding these elements makes placements more accessible and genuinely transformative, particularly for students from underrepresented backgrounds, who often benefit most from consistent mentoring and early professional identity-building experiences.

### Assess work-based learning

To ensure placements are academically rigorous and pedagogically valuable, they must be formally assessed. Assessment validates the learning, encourages reflection, and ensures accountability on the part of students, employers, and universities alike (49).

A variety of assessment methods can be employed such as (a) reflective journals or e-portfolios to promote self-evaluation (50, 51), (b) supervisor evaluations to assess professionalism and skill development (52), (c) work placement reports or presentations linking experience to academic theory and (53) (d) peer or group reflections to foster critical thinking and knowledge sharing (54).

These assessments should be credit-bearing and integrated into the programme structure. They provide students with tangible outputs that can be used in job applications and interviews, while also giving staff and stakeholders insight into placement quality and learning outcomes. A strong assessment strategy also ensures parity with other modules and legitimizes work based learning as an essential part of the academic journey.

### Policy reforms for inclusive research and student placements

Research funding bodies should embed equity and inclusion more explicitly into grant requirements by requiring funded projects to offer structured opportunities for undergraduate involvement, particularly for students from underrepresented backgrounds. Building on UKRI’s existing expectations around public engagement and equality, diversity and inclusion (EDI), such measures would provide students with early research experience, mentoring, and recognition, while diversifying the research pipeline (55). Complementary policies could include tax incentives or grants for employers offering quality placements, reserved bursaries to support marginalized students, and incorporating placement outcomes into institutional performance metrics. Together, these changes would align educational, research, and inclusion priorities, ensuring placements and research opportunities are both accessible and transformative.

## Conclusion

While learning for its own sake remains a core academic value, contextualizing theoretical knowledge through work-integrated learning enhances understanding, even for those pursuing education solely for intellectual development. Expanding access to such experiences strengthens engagement and real-world relevance without compromising academic goals. Embedding structured, equitable, and assessed work experience into non-accredited science degrees is essential for preparing graduates who are workforce-ready and socially mobile. Leaving placements to student initiative reinforces inequality and restricts opportunities for underrepresented students. This Perspectives paper highlights the need for universities to take institutional responsibility for fair access to work-integrated learning.

## Data Availability

The original contributions presented in this study are included in this article/supplementary material, further inquiries can be directed to the corresponding author.
